# NMR Characterization of Ten Apple Cultivars from the Piedmont Region

**DOI:** 10.3390/foods10020289

**Published:** 2021-02-01

**Authors:** Giacomo Di Matteo, Mattia Spano, Cristina Esposito, Cristina Santarcangelo, Alessandra Baldi, Maria Daglia, Luisa Mannina, Cinzia Ingallina, Anatoly P. Sobolev

**Affiliations:** 1 Department of Chemistry and Technology of Drugs, Sapienza University of Rome, Piazzale Aldo Moro 5, 00185 Rome, Italy; giacomo.dimatteo@uniroma1.it (G.D.M.); mattia.spano@uniroma1.it (M.S.); luisa.mannina@uniroma1.it (L.M.); 2 Department of Pharmacy, University of Naples Federico II, 80138 Naples, Italy; cristina.esposito@unina.it (C.E.); cristina.santarcangelo@unina.it (C.S.); maria.daglia@unina.it (M.D.); 3 Tefarco Innova, Parco Area delle Scienze 27/A—Campus, 43124 Parma, Italy; alessandra.baldi.alimenti@gmail.com; 4 International Research Center for Food Nutrition and Safety, Jiangsu University, Zhenjiang 212013, China; 5 Institute for Biological Systems, Magnetic Resonance Laboratory “Segre-Capitani”, CNR, Via Salaria Km 29.300, 00015 Monterotondo, Italy; anatoly.sobolev@cnr.it

**Keywords:** *malus domestica*, local apple cultivars, NMR, metabolite profile, PCA, food ingredients

## Abstract

The metabolite profile of ten traditional apple cultivars grown in the Piedmont region (Italy) was studied by means of nuclear magnetic resonance spectroscopy, identifying an overall number of 36 compounds. A more complete assignment of the proton nuclear magnetic resonance (^1^H NMR) resonances from hydroalcoholic and organic apple extracts with respect to literature data was reported, identifying fructose tautomeric forms, galacturonic acid, γ-aminobutyric acid (GABA), *p*-coumaroyl moiety, phosphatidylcholine, and digalactosyldiacylglycerol. The chemical profile of each apple cultivar was defined by thorough quantitative NMR analysis of four sugars (fructose, glucose, sucrose, and xylose), nine organic acids (acetic, citric, formic, citramalic, lactic, malic, quinic, and galacturonic acids), six amino acids (alanine, asparagine, aspartate, GABA, isoleucine, and valine), rhamnitol, *p*-coumaroyl derivative, phloretin/phloridzin and choline, as well as β-sitosterol, fatty acid chains, phosphatidylcholine, and digalactosyldiacylglycerol. Finally, the application of PCA analysis allowed us to highlight possible differences/similarities. The Magnana cultivar showed the highest content of sugars, GABA, valine, isoleucine, and alanine. The Runsé cultivar was characterized by high amounts of organic acids, whereas the Gamba Fina cultivar showed a high content of chlorogenic acid. A significant amount of quinic acid was detected in the Carla cultivar. The knowledge of apple chemical profiles can be useful for industries interested in specific compounds for obtaining ingredients of food supplements and functional foods and for promoting apple valorization and preservation.

## 1. Introduction

Apples are the third most produced fruits in the world [1] after bananas and watermelons, consumed in the human diet as raw or dried products, juice, paste, jam and syrups. From a nutritional point of view, apples are a low-fat (less than 1%) and low-protein food (less than 1%) [2], whereas sugars represent about 10% of total apple weight, with fructose being the most abundant sugar (6%). Micronutrients, such as minerals (mainly potassium, phosphorus, calcium, and magnesium), vitamins (mainly vitamin C), and secondary metabolites (such as phenolic compounds [3,4]) are also present. Moreover, apples represent an important source of pectin, a gelling agent obtained from apple pomaces, used for many food industrial productions [5,6].

In 2018, more than 86 million tons of apples were produced worldwide, with China being the main producer (39 million tons). Italy is the sixth largest producer in the world with 2.4 million tons in 2018 [1]. In many Italian regions, local apple cultivars, used and consumed by the local population, represent an important crop, especially in terms of the local economy. In particular, Alto Adige (912.757 tons in 2020) and Trentino (496.783 tons in 2020) are the most important growing areas of high-quality apples throughout Europe, despite the fact that the cultivation area is not particularly large. However, Piedmont represents today the third largest Italian producer, contributing 225.281 tons of apples to the national production and with an increase of +13% with respect to 2019. Indeed, although Piedmont apple cultivation is not well-known, the story of Piedmont apple growing dates back to the Middle Ages, when the monastic orders cultivated and improved the varieties that had survived barbarian invasions [7,8,9]. By the early 20th century, Piedmont was home to thousands of varieties, but with the advent of industrial agriculture, decisions with great impact were made regarding selection. The market preferred more productive and attractive apple cultivars, characterized by a bigger size, more attractive appearance, and less delicate flavor, leading to the loss of traditional Italian apple cultivars [10], which survived only in local and niche areas. Today, a turnaround can be observed—the rediscovery of traditional and ancient cultivars that often represent more environmentally sustainable cultivation than commercial ones [11], maintaining both biodiversity and the historical and cultural links [12]. In this scenario, deep characterization and valorization of this heritage is fundamental in order to preserve the special quality of these fruits and to avoid the loss of precious and useful germplasms [12].

Apples’ flavor, consistency, taste and, health-related properties strictly depend on the fruit’s chemical composition and on the balance between apple component levels. For instance, each organic acid gives rise to a particular acidity sense, and each sugar has its own sweetness level. Apple chemical composition has been largely investigated using targeted chromatographic techniques, such as high-performance liquid chromatography (HPLC) to determine phenolic content [13,14,15,16] and gas chromatography (GC) for the determination of sugars, polyols, sugar phosphates, organic acids, sterols, polyphenols [17], fatty acids [18], and other aromatic compounds [19,20]. Untargeted proton nuclear magnetic resonance (^1^H NMR) methodologies [21] have focused mainly on apple juice matrixes [19,22,23] to study the chemical composition of new cultivars [24], cultivars with different resistances against fungi attacks [25], and commercial cultivars from Japan and New Zealand [26]. ^1^H high field NMR spectroscopy has already shown to be a valuable tool to investigate local typical products, such as red sweet peppers [27], white celery [28], tomatoes [29], and olive oils [30]. To date, ancient apple cultivars from Piedmont have been investigated in term of sensory parameters, nutritional aspects [12,31], and genetic characterization [32] through the application of standard analytical techniques. Both Donno et al. and Contessa et al. highlighted superior nutritional traits of ancient Piedmont apple cultivars, with a generally high content of organic acids, sugars, and total phenolic compounds [31]. However, deep chemical characterization by means of advanced analytical methodologies has never been performed. 

In this paper, the hydroalcoholic and organic extracts of ten traditional apple cultivars grown in the Piedmont region (northwest Italy) were investigated by means of the NMR methodology to determine their apple metabolite profiles. The application of principal component analysis (PCA) to NMR data allowed us to summarize and highlight the differences and similarities between the selected apple cultivars. Indeed, mathematical tools, better known as chemometrics, are widely used for metabolomic data analysis. In particular, unsupervised methods are utilized to summarize, explore, and discover clusters or trends in the data without a priori attribution of any class membership [33]. 

The knowledge of chemical apple profile can be extremely important for the introduction of local products in national and international markets and for industries interested in specific apple compounds.

## 2. Materials and Methods

### 2.1. Sampling

**Figure 1:**
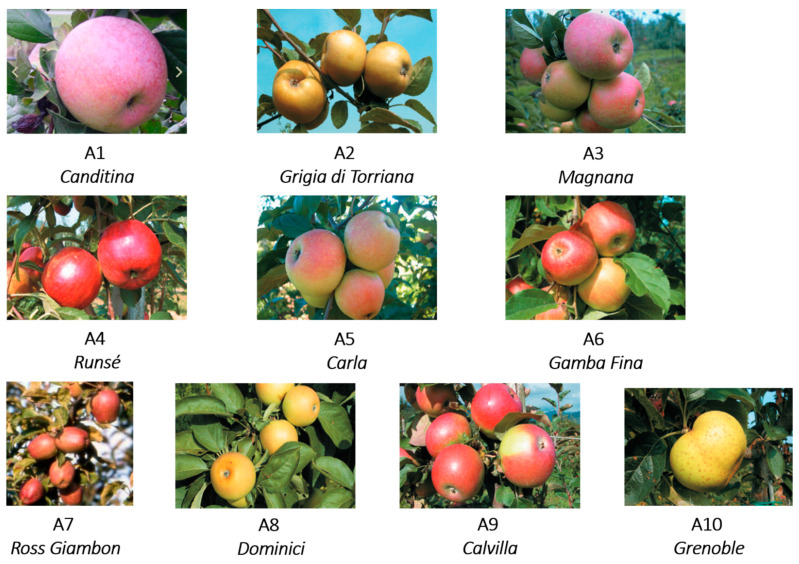
Apple cultivars from the Piedmont region.

Ten apple cultivars (*Malus domestica*) typical of Piedmont region, Italy (Figure 1) were collected in the period of their complete maturity, specific for every cultivar, as reported in Table 1. The apples were provided by three farms: “Azienda Agricola Melamangio”, “Scuola Malva Arnaldi”, and “Azienda Agricola Turaglio”. In particular the “Azienda Agricola Melamangio” farm, located in Odalengo Piccolo, provided the variety Canditina (A1); “Scuola Malva Arnaldi”, located in Bibiana, provided five varieties: Grigia di Torriana (A2), Magnana (A3), Runsé (A4), Carla (A5) and Gamba Fina (A6); “Azienda Agricola Turaglio”, located in Cavour, provided four varieties: Ross Giambon (A7), Dominici (A8), Calvilla (A9) and Grenoble (A10). Some features of the ten cultivars, such as maturity time, size, peel tactility and color, pulp consistency, and color, are reported in [7,8,9].

### 2.2. Chemicals

Deuterated water (D_2_O) 99.97% D, methanol-D4 99.80% D, chloroform-D 99.80% D + 0.03% tetramethylsilane (TMS), and 3-(trimethylsilyl)-propionic-2,2,3,3-d4 acid sodium salt (TSP) were purchased from Euriso–Top (Saclay, France). Anhydrous potassium phosphate dibasic (K₂HPO₄) and anhydrous potassium phosphate monobasic (KH₂PO₄) were purchased from Sigma–Aldrich (St. Louis, MO, USA). Methanol (HPLC-grade) and chloroform (HPLC-grade) were purchased from Carlo Erba Reagenti (Milan, Italy). Millipore grade water was purchased from Tito Menichelli S.r.l. (Rome, Italy).

### 2.3. Sample Preparation

In the sample preparation step, apple skin and pulp and seeds were removed. Seven apples were sampled for each cultivar. The apples were first washed with water and Na_2_CO_3_ to eliminate every dirt residue. A clove was taken from each apple and subsequently shredded with a ceramic knife. To control the oxidation during preparation, the samples were cut in an ice bath. All the samples were transferred to the falcon and lyophilized for 7 days. All apples were cut into eight parts, and seven pieces (one from each apple of the cultivar) were chopped together into smaller pieces. Each sample was then freeze-dried and pulverized with mortar and pestle. The freeze-drying process promotes water removal, thereby reducing the oxidation process. 

### 2.4. Extraction Procedure for NMR Analysis

Extracts for NMR analysis were obtained following the protocol previously described [35] with some modifications. Powder (0.5 g) was sequentially added with a 3 mL methanol/chloroform (2:1 *v*/*v*) mixture, containing 1 mL of chloroform and 1.8 mL of Millipore-grade water, which was shaken slightly after each solvent addition. The obtained emulsion was stored at 4 °C for 40 min and then centrifuged at 4200× *g* for 15 min at 4 °C. The organic and hydroalcoholic phases were separated, and the residual pellet was extracted again using half of the solvent volumes to ensure complete extraction of the soluble metabolites. A slow N_2_ flow was used for drying. The obtained extracts were stored at −20 °C until analysis. 

### 2.5. Metabolic Profile by NMR Analysis

Hydroalcoholic and organic extracts were analyzed using a Bruker AVANCE 600 spectrometer (Bruker, Milan, Italy) at 28 °C operating at 600.13 MHz (proton frequency) and equipped with a Bruker multinuclear z-gradient 5 mm probe head. The temperature for NMR spectral analysis (28 °C) was chosen according to the internal laboratory protocol that assures temperature maintenance within (±0.1 °C) limits and is close to the experimental conditions reported in most databases. Each dried hydroalcoholic extract was solubilized in 1 mL of D_2_O; 0.2 mL of the obtained solution was mixed with 0.5 mL of 400 mM phosphate buffer (pH 7.4)/D_2_O containing 2 mM solution of TSP (internal standard) and transferred into a 5 mm NMR tube. The use of TPS as internal standard does not interfere with the protein in apple extracts, as the balk of apple proteins is not easily soluble in water [36]. ^1^H spectra (Bruker pulse sequence *zgpr*) of hydroalcoholic extracts were acquired with 200 transients, a recycle delay of 5 s, an acquisition time of 2.28 s, a 90° flip angle pulse of 14 µs, and 32K data points. The water signal was suppressed using solvent pre-saturation. Each dried organic extract was solubilized in 0.7 mL of the CDCl_3_/CD_3_OD (2:1 *v*/*v*) mixture and transferred into a 5 mm NMR tube that was flame sealed. ^1^H spectra (Bruker pulse sequence *zg*) of the organic extracts were acquired with 128 transients, a recycle delay of 5 s, an acquisition time of 1.82 s, a 90° flip angle pulse of 10.5 µs, and 32K data points. 

Two-dimensional (2D) NMR experiments (^1^H-^1^H TOtal Correlated SpectroscopY (TOCSY), ^1^H-^13^C Heteronuclear Single Quantum Coherence (HSQC), and ^1^H-^13^C Heteronuclear Multiple Bond Correlation (HMBC) were performed on each hydroalcoholic and organic extract under the experimental conditions previously reported [29].

In order to evaluate the repeatability of the protocol and to ensure the complete extraction of the soluble metabolites, all dry matter after freeze-drying was recovered, and the entire procedure (from the extraction to NMR analysis) was carried out in triplicate. 

In Table 2, the signals of the identified metabolites in the hydroalcoholic extracts ^1^H NMR spectra are reported. Among them, the 26 selected signals marked with asterisks were integrated using the Bruker TOPSPIN 1.3 software and normalized with respect to the methyl group signal of TSP (0.00 ppm), set to 100. The quantified metabolites were expressed in mg/100 g of the dried sample ± SD (standard deviation) (Table S1). 

The integral areas of the 7 selected signals in the ^1^H NMR spectra of organic extracts (Table 3) were measured using the Bruker TOPSPIN 1.3 software and normalized with respect to the integral (I_FA_) of the α-CH_2_ group signal of all fatty acids (2.30 ppm), set to 100. The molar % values ± SD of fatty acids, β-sitosterol, phosphatidylcholine, and digalactosyldiacylglycerol were calculated with consideration of the number of equivalent protons using the following equations:

%_STE_ = 100(0.66I_STE_/I_tot_) (1)

%_TRI_ = 100(0.5I_TRI_/I_tot_) (2)

%_DI_ = 100(I_DI_/I_tot_) (3)

%_MONO_ = 100(I_UNS_ − 2I_DI_ − 1.5I_TRI_)/I_tot_ (4)

%_SAT_ = 100(I_FA_ − I_DI_ − 0.5I_TRI_ − %_MONO_)/I_tot_ (5)

%_PC_ = 100(4I_PC_/9I_tot_) (6)

%_DGDG_ = 100(4I_DGDG_/I_tot_) (7)

where %_STE_, %_TRI_, %_DI_, %_MONO_, %_SAT_, %_PC_, and %_DGDG_ are the molar % of β-sitosterol, tri-unsaturated fatty acids, di-unsaturated fatty acids, mono-unsaturated fatty acids, saturated fatty acids, phosphatidylcholine, and digalactosyldiacylglycerol, respectively. I_STE_, I_TRI_, I_DI_, I_UNS_, I_FA_, I_PC_, and I_DGDG_ are integrals, whereas I_tot_ is calculated according to the following equation:

I_tot_ = I_FA_ + 0.66I_STE_ (8)

The amount of each quantified metabolite in organic extracts is reported in Table S2.

### 2.6. Multivariate Statistical Analysis

Principal component analysis (PCA) was carried out on 30 selected variables corresponding to 23 metabolites from the hydroalcoholic extract and 7 from the organic extract. In the case of fructose, glucose, and xylose, the sum of their alpha and beta anomeric forms instead of the individual isomer content was used in statistics. Before statistical analysis, the data were preprocessed using autoscaling: all of the variables were mean centered, and each variable was divided by its standard deviation. Principal component analysis was carried out using SIMCA software (version 12).

## 3. Results

### 3.1. Assignments of Aqueous and Organic Extracts

**Figure 2:**
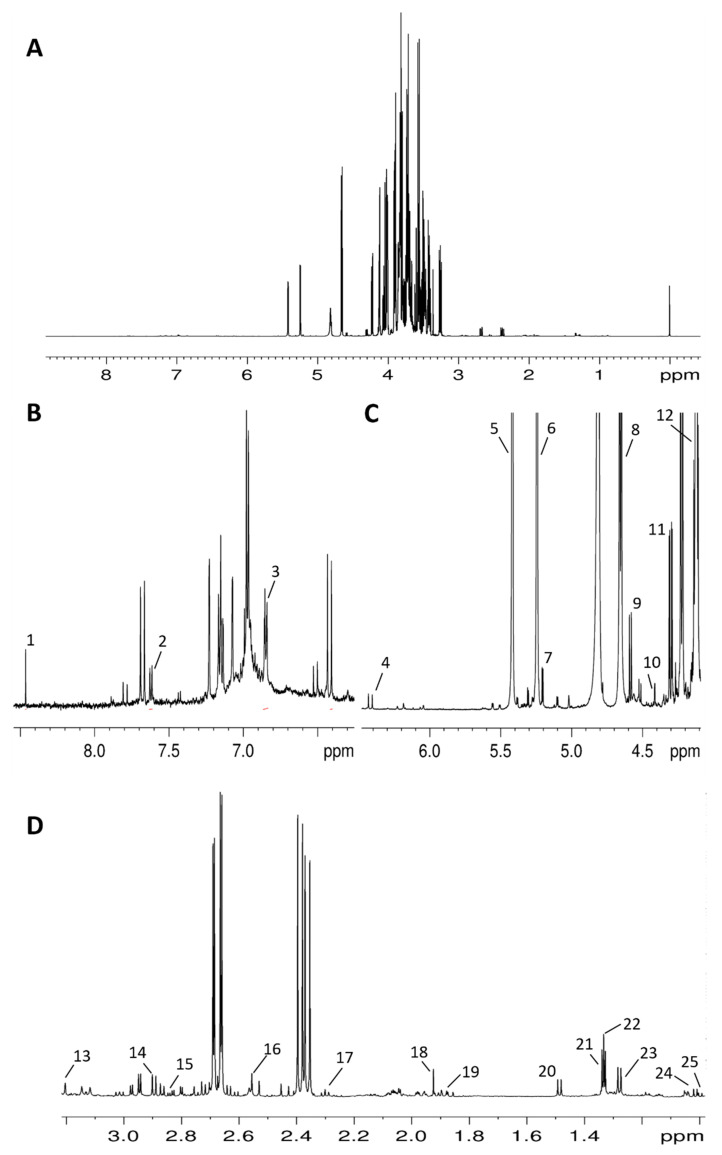
600 MHz ^1^H NMR spectrum of hydroalcoholic extract from apple fruit (var. Magnana) in a 400 mM phosphate buffer (pH 7.4)/D_2_O mixture with 2mM of 3-(trimethylsilyl)-propionic-2,2,3,3-d4 acid sodium salt (TSP). (**A**) Entire spectrum. (**B**) Low field region: 1, formic acid; 2, *p*-coumaric acid derivative; 3, phloretin/phloridzin. (**C**) Middle field region: 4, chlorogenic acid; 5, sucrose; 6, α-glucose; 7, α-xylose; 8, β -glucose; 9, β-xylose; 10, α-galacturonic acid; 11, malic acid; 12, D-fructofuranose, (**D**) High field region**:** 13, choline; 14, aspartate; 15, asparagine; 16, citric acid; 17, γ-aminobutyrate; 18, acetic acid; 19, quinic acid; 20, alanine; 21, lactic acid; 22, citramalic acid; 23, rhamnitol; 24, isoleucine; 25, valine.

**Figure 3:**
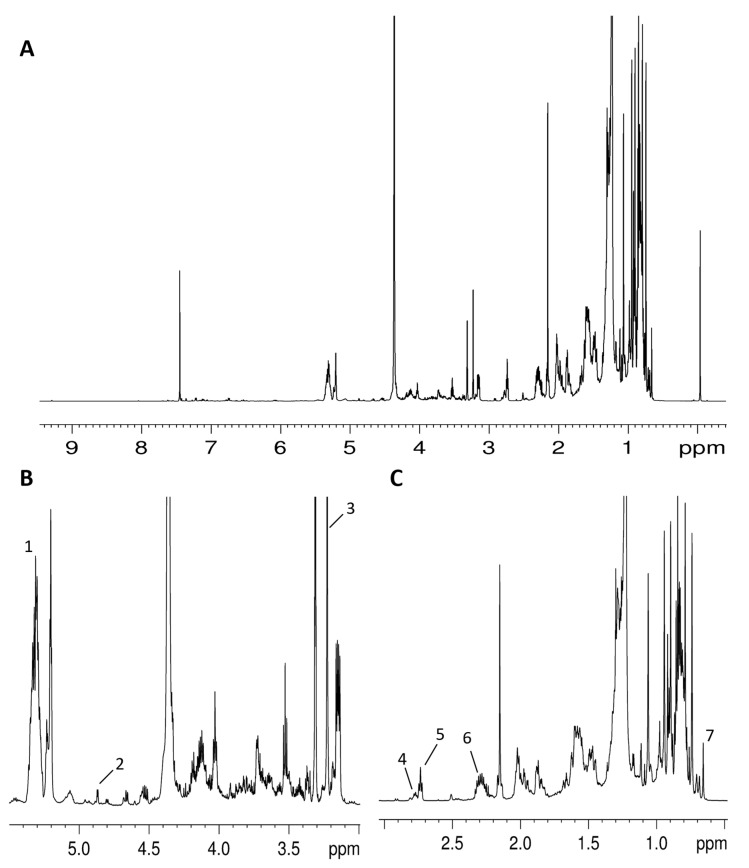
^1^H NMR spectrum at 600 MHz of organic extract from apple fruit (var. Magnana) in the CDCl_3_/CD_3_OH 2:1 (*v*/*v*) mixture. (**A**) Entire spectrum. (**B**) Middle field region: 1, total unsaturated fatty acids; 2, digalactosyldiacylglycerol; 3, 1,2-diacyl-*sn*-glycero-3-phosphatidylcholine. (**C**) High field region**:** 4, linolenic fatty chain; 5, linoleic fatty chain; 6, total fatty acids; 7, β-sitosterol.

The ^1^H and ^13^C NMR spectra assignments apple aqueous (Figure 2) and organic (Figure 3) extracts of apple were carried out using 2D NMR experiments, standard compound addition, and literature data [24,25,26,37]. A more complete spectral assignment (Table 2) of the aqueous extracts with respect to literature data was obtained, identifying fructose tautomeric forms, galacturonic acid, GABA, and *p*-coumaroyl moiety. Fructose tautomeric forms, namely α-D-fructofuranose and β-D-fructopyranose, were identified by means of their diagnostic ^1^H NMR signals and 2D experiments. In particular, the presence of α-D-fructofuranose was suggested by its characteristic signal at 4.13 ppm due to the CH-3 proton. ^1^H-^1^H TOCSY experiment allowed us to identify the correlation with the CH-4 proton (4.00 ppm), whereas the ^1^H-^13^C HMBC experiment showed the correlation of the CH-3 proton with the C-2 carbon at 105.5 ppm, typical of ketoses. Analogously, β-D-fructopyranose was recognized by the signal of the CH-5 proton at 4.01 ppm, and the diagnostic spin system detected by the ^1^H-^1^H TOCSY experiment allowed us to identify the other protons of this sugar, namely CH-3 (3.80 ppm), CH-4 (3.90 ppm), and CH_2_-6,6′ (3.72, 4.03 ppm). C-2 carbon at 99.2 ppm was also identified by means of the ^1^H-^13^C HMBC map. The presence of α-galacturonic moiety was suggested by the diagnostic chemical shifts of the spin systems in the ^1^H-^1^H TOCSY experiment, the coupling constants, and the correlations in the ^1^H-^13^C HMBC experiment. In particular, the doublet at 4.41 ppm with a J coupling constant of 1.2 Hz due to CH-5 proton indicates single coupling between H-5 and the equatorial H-4. The ^1^H-^1^H TOCSY experiment allowed us to identify the other protons of this metabolite, namely CH-4 (4.29 ppm), CH-3 (3.90 ppm), CH-2 (3.80 ppm), and CH-1 (5.31 ppm, doublet with J = 3.80 Hz). The correlation of the CH-5 proton with carboxylic carbon at 177.1 ppm was confirmed by the ^1^H-^13^C HMBC map. 

The presence of GABA was confirmed by its typical triplet at 2.30 ppm (*J* = 7.4 Hz) assigned to α-CH_2_ protons. The diagnostic spin systems identified in the ^1^H-^1^H TOCSY experiment allowed us to observe the correlations with β-CH_2_ and γ-CH_2_ protons at 1.90 and 3.01 ppm, respectively.

The diagnostic spin systems identified in the ^1^H-^1^H TOCSY experiment suggested the presence of *p*-coumaroyl moiety due to the correlation between H-2/H-6 equivalent aromatic signals at 7.62 ppm and H-3/H-5 equivalent signals at 6.97 ppm (*J* = 8.8 Hz corresponding to *ortho* position) and the correlation between the double bond protons at 7.79 and 6.51 ppm with *J* = 16.1 Hz corresponding to *trans* configuration of the double bond. The low intensity of these signals—and, therefore, the low concentration of the corresponding compound—does not allow further correlations to be observed in the 2D experiments to complete the structural assignment. *p*-Coumaroyl quinic acid is the principal *p*-coumaroyl ester previously identified in apples using the targeted HPLC methodology [13,38,39]. Therefore, the identified *p*-coumaroyl moiety can be bound to quinic acid; however, the esterified quinic acid moiety was not detected due to the signal overlapping.

It was not possible to distinguish phloretin from its glycosides (such as phloridzin) using the available signals from aromatic protons region. Unfortunately, the signals expected for β-glucose moiety were not observed due to the strong overlapping with markedly more intense signals of other carbohydrates. The *orto*- and *meta*- protons of the *para*-substituted aromatic ring, a common moiety for all phloretin derivatives, gave rise to the signals at 7.16 and 6.85 ppm, respectively.

In Table 2, the assignment of four sugars (fructose, glucose, sucrose, and xylose), nine organic acids (acetic, citric, formic, citramalic, lactic, malic, quinic, and galacturonic acids), six amino acids (alanine, asparagine, aspartate, GABA, isoleucine, and valine), rhamnitol, *p*-coumaroyl derivative, phloretin/phloridzin, and choline is reported.

A more complete assignment of organic extracts of apples (Table 3) with respect to the literature data was obtained, reporting the assignment of glycerogalactolipid and glycerophospholipid polar heads. The presence of digalactosyldiacylglycerol (DGDG), the most abundant galactolipid in apples [40], was suggested by the characteristic doublet (*J* = 3.8 Hz) at 4.87 ppm due to the equatorial CH’’-1 proton of the external galactose ring [41]. The ^1^H-^1^H TOCSY experiment allowed us to identify other protons of the ring, namely CH-2” (3.77 ppm), CH-3” (3.69 ppm), and CH-4” (3.91 ppm). Analogously, the CH-1′ proton of the internal DGDG galactose was also assigned at 4.19 ppm, together with the CH-2′/CH-3′ and CH-4′ protons (3.50–3.53 and 3.90 ppm, respectively).

Methyl groups of phosphatidylcholine N^+^-(CH_3_)_3_ moiety were detected by the diagnostic ^1^H and ^13^ C signals at 3.22 ppm at 54.5 ppm, respectively. Moreover, the ^1^H-^1^H TOCSY experiment allowed us to identify proton correlation between the CH_2_OP group at 4.45 ppm (^13^C 60.6 ppm) and the CH_2_N^+^ protons at 3.75 ppm (^13^C 66.4 ppm).

In Table 3, the assignment of β-Sitosterol, fatty acid chains, phosphatidylcholine, and digalactosyldiacylglycerol is also reported. 

### 3.2. Metabolite Profiles of Apple Cultivars 

In this section, each class of compounds is discussed separately, and a comparison of the ten cultivars is conducted.

#### 3.2.1. Sugars and Polyols

**Figure 4:**
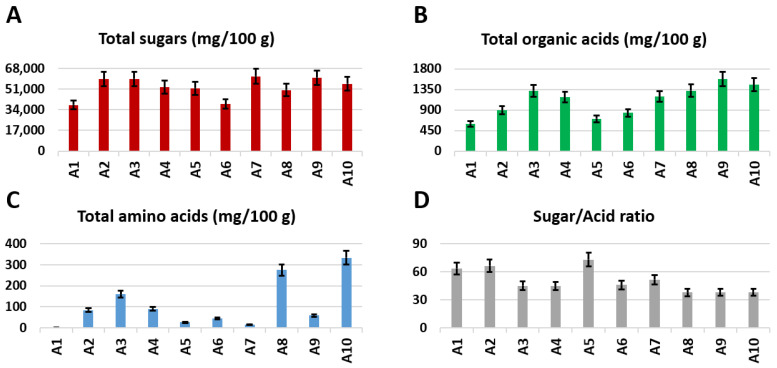
Bar charts of the total content of (**A**) sugars, (**B**) organic acids, (**C**) amino acids, (**D**) sugar/acid ratio, identified and quantified (mg/100 g of DW ± SD) in the ^1^H NMR spectra of hydroalcoholic extracts of apples.

**Figure 5:**
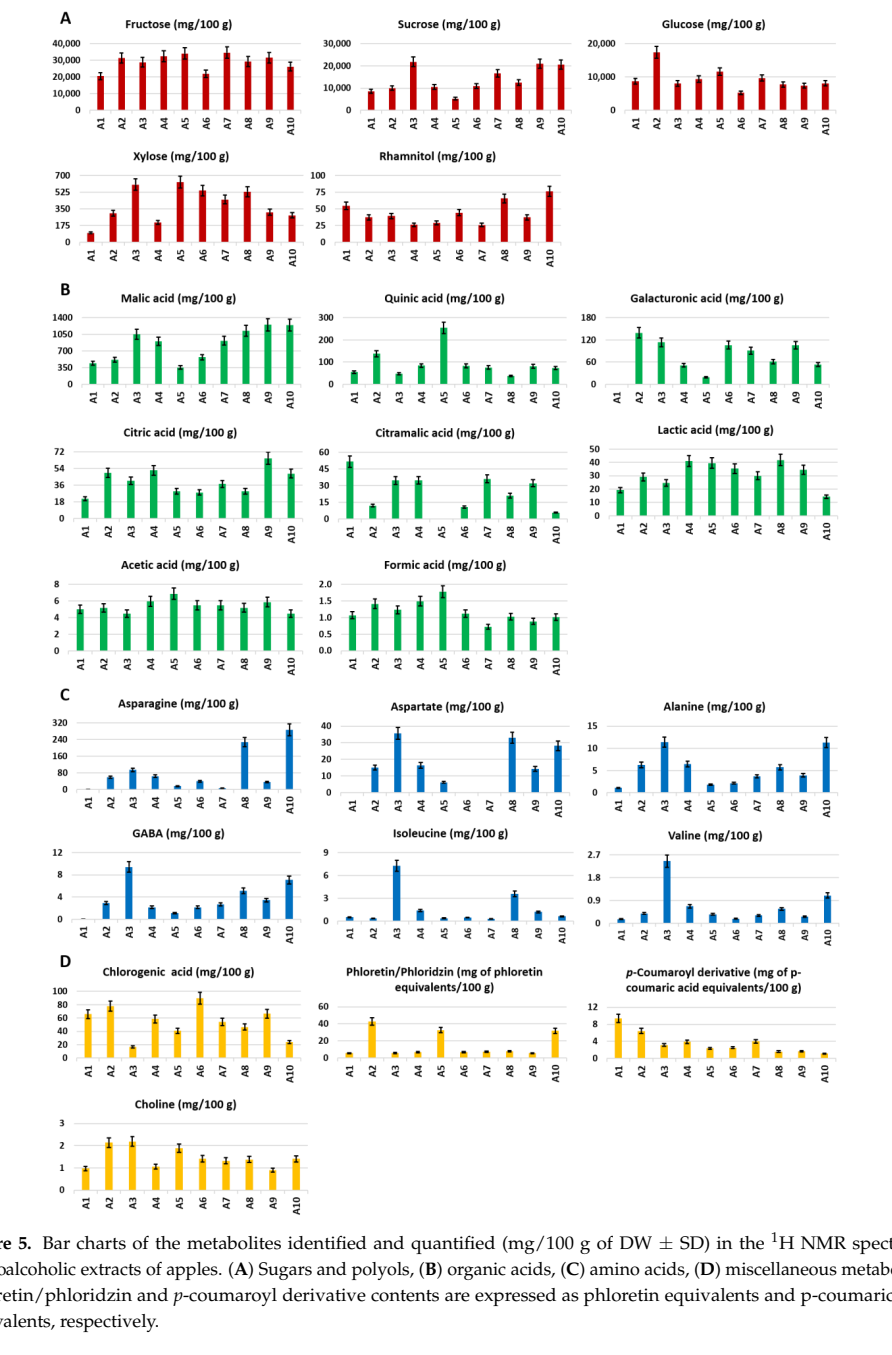
Bar charts of the metabolites identified and quantified (mg/100 g of DW ± SD) in the ^1^H NMR spectra of hydroalcoholic extracts of apples. (**A**) Sugars and polyols, (**B**) organic acids, (**C**) amino acids, (**D**) miscellaneous metabolites. Phloretin/phloridzin and *p*-coumaroyl derivative contents are expressed as phloretin equivalents and p-coumaric acid equivalents, respectively.

The highest content of total sugars was measured in cultivars A9, A7, A3, and A2, whereas the lowest content was found in cultivars A1 and A6 (Figure 4). In particular, fructose, glucose and, sucrose were the most abundant sugars in all apple cultivars (Figure 5A). As expected [18], fructose turned out to be the main sugar in all samples, with the highest content detected in cultivars A5 and A7 and the lowest content in A1. The highest value of glucose content was measured in cultivar A2, whereas the lowest content ( more than three times less) was identified in sample A6. According to literature data [18,42,43], the fructose-to-glucose ratio was higher than 1.7 in all of the analyzed samples. Moreover, the fructose/glucose, fructose/sucrose, and sugar/acid weight ratios were calculated (Table 4). Cultivar A3 showed the highest sucrose content, whereas the lowest content was observed in A5 (more than four times less). Xylose was present in low concentrations in all of the cultivars, with cultivar A5 showing the highest content, whereas A1 showed the lowest content (more than six times). The highest content of rhamnitol, a polyol, was measured in cultivar A10, whereas the lowest content was found in A7. 

#### 3.2.2. Organic Acids

The highest total organic acids content (Figure 4) was found in cultivars A9 and A10, whereas the lowest amount was observed in cultivar A1 (more than two times less). In particular, malic acid was the most abundant acid in apples. The highest concentrations of malic acid were found in samples A9 and A10, whereas the lowest concentration was observed in A5 (Figure 5B). Cultivar A5 presented by far the highest content of quinic acid, whereas the lowest content (almost seven times less) was found in sample A8. The highest content of galacturonic acid was measured in cultivar A2, whereas it was not detected in sample A1. Cultivar A9 showed the highest amount of citric acid, whereas the lowest content was measured in A1 (more than three times less). Citramalic acid was not detected in sample A5; conversely, the highest amount was measured in sample A1. Cultivars A4 and A8 showed the highest content of lactic acid, whereas the lowest content was found in A10. Sample A10 was also characterized by the lowest amount of acetic acid, whereas cultivar A5 showed the highest amount. Finally, formic and acetic acids were found in very low concentrations, below 2 mg/100 g.

#### 3.2.3. Amino Acids

The content of free amino acids (total and those of individual components) was extremely variable among the different cultivars (Figure 5C). The highest total amino acid content was found in cultivars A8 and A10, whereas the lowest amount was observed in cultivars A1, A7, and A5 (Figure 4). In particular, cultivar A3 showed the highest amount of GABA, valine, and isoleucine, and, together with cultivar A10, alanine. Asparagine content varied from 280 mg/100 g in A10 to less than 1 mg/100 g (below the limit of detection) in A1. Aspartate was not found in cultivars A1, A6, or A7, and GABA was not detected in cultivar A1.

#### 3.2.4. Miscellaneous

Cultivar A6 showed the highest amount of chlorogenic acid, whereas cultivar A3 the lowest amount (5 times less). A2, A5, and A10 showed by far the highest amounts of phloretin/phloridzin, whereas the lowest amount was observed in cultivar A1. The highest amount of *p*-coumaroyl derivatives was detected in cultivar A1, whereas the lowest amount was found in A10. The highest amount of choline was measured in cultivars A2 and A3, whereas the lowest amount was found in samples A1 and A9 (Figure 5D). 

#### 3.2.5. β-Sitosterol

**Figure 6:**
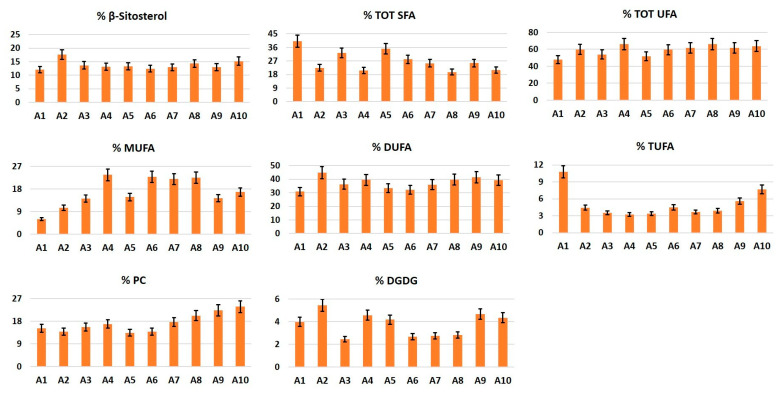
Bar charts of the metabolites (molar % ± SD) identified and quantified in the ^1^H NMR spectra of organic extracts of apples.

This molecule was found in appreciable concentrations in all of the analyzed cultivars. In particular, the highest value was measured in cultivar A2, whereas the lowest amount was identified in A1 (Figure 6).

#### 3.2.6. Fatty Acids

The content of unsaturated fatty acids (TOT UFA) was higher than the content of saturated fatty acids (TOT SFA) in all of the ten cultivars. Di-unsaturated fatty acids (DUFA) were the most abundant unsaturated fatty acids in all of the samples, the highest level being measured in cultivar A2 and the lowest in A1. Sample A1 was also characterized by the lowest value of mono-unsaturated fatty acids (MUFA) and the highest value of tri-unsaturated fatty acids (TUFA). Cultivar A4 showed an opposite trend, having the highest amount of MUFA and the lowest amount of TUFA. Finally, the highest concentration of TOT SFA was found in cultivar A1, whereas the lowest concentration was measured in sampleA8 (Figure 6).

#### 3.2.7. Polar Lipids

The highest content of phosphatidylcholine (PC) was observed in cultivar A10, whereas the lowest content was measured in A5. Cultivar A2 showed the highest concentration of digalactosyldiacylglycerol (DGDG), whereas the lowest content was observed in sample A3 (Figure 6). 

### 3.3. Multivariate Statistycal Analysis (PCA)

**Figure 7:**
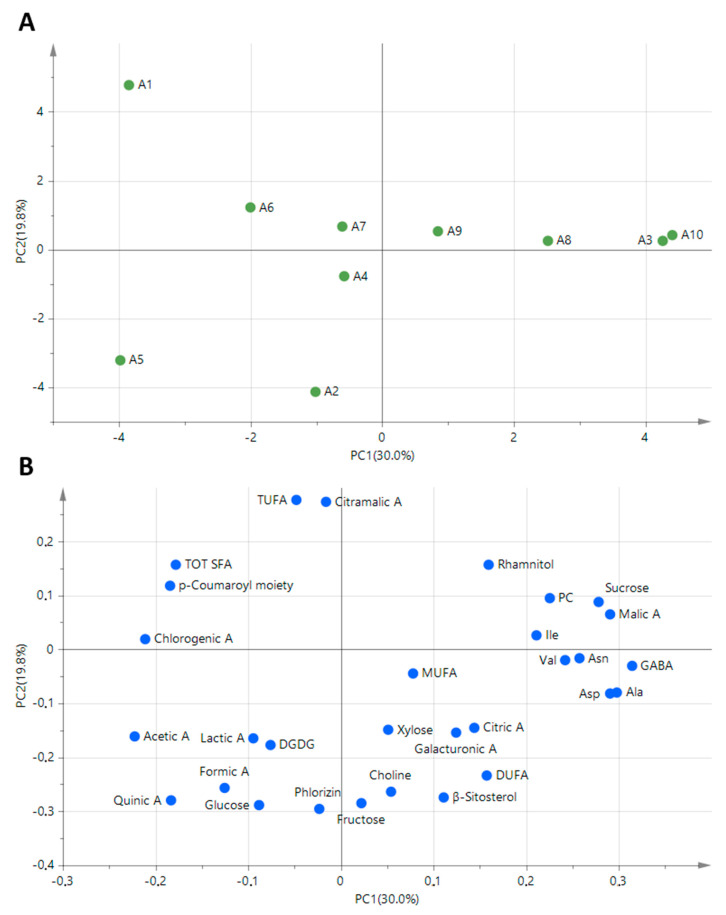
PCA applied to NMR data of the ten apple cultivars. (**A**) Sample scores and (**B**) loadings. PC1 and PC2 represent 30.0% and 19.8% of the total variance, respectively.

The overall analysis of the histograms made it possible to find characteristic features relative to each cultivar. Moreover, PCA applied to all of the NMR variables allowed us to highlight possible cultivar similarities and differences (Figure 7). 

Cultivar A1 (Canditina) was observed to be well separated from all of the others for the high content of citramalic acid, *p*-coumaroyl moiety, TUFA, and TOT SFA, and low amounts of xylose, fructose, sucrose, malic acid, citric acid, alanine, and MUFA. Notably, galacturonic acid, asparagine, aspartate, and GABA were not detected. 

Carla, cultivar A5, was characterized by high contents of fructose, xylose, and organic acids (such as quinic acid, lactic acid, acetic acid, and formic acid) and low amounts of sucrose, chlorogenic acid, malic acid, amino acids (namely, asparagine aspartate, and alanine), and malic and galacturonic acids. Citramalic acid was not detected.

Gamba Fina, cultivar A6, was characterized by high content of MUFA and chlorogenic acid and low amounts of fructose, glucose, and citramalic acid. Aspartate was not detected.

Ross Giambon, cultivar A7, was characterized by high fructose and MUFA contents and low amounts of rhamnitol and asparagine. The level of aspartate was beyond the limit of detection.

Runsé, cultivar A4, was characterized by high amounts of organic acids and MUFA.

Grigia di Torriana, cultivar A2, showed high amounts of glucose, galacturonic acid, choline, DUFA, and DGDG and a low level of citramalic acid.

Calvilla, cultivar A9, was characterized by high levels of sucrose, organic acids, DUFA and PC.

Dominici, cultivar A8, showed high contents of rhamnitol, lactic acid, asparagine, aspartate, isoleucine, and MUFA and a low concentration of quinic acid. 

The Magnana (A3) and Grenoble (A10) cultivars, situated nearby on the PCA score plot (PC1 > 4, Figure 3a), were characterized by high levels of sucrose, rhamnitol, malic acid, asparagine, alanine, and PC and low content of *p*-coumaroyl moiety.

## 4. Discussion

The present data on the chemical composition of traditional apple cultivars of the Piedmont region can be compared with those of some of the most widely cultivated apple cultivars in the world [46,47], such as Golden Delicious, Fuji, Granny Smith, and Jonagold [18,44,45]. Taking into account the fact that the data reported in the literature are related to apple juice composition (usually expressed as g/L or g/kg FW), while our data are related to dried tissue (expressed as mg/100 g of DW), it is clear that only the ratios between the components and not the absolute values can be directly compared. 

The sugar content in apple fruit is very important, especially for diabetic patients who adapt their insulin intake in relation to the carbohydrate content of each food. Table 4 shows the ratios of fructose/glucose and fructose/sucrose calculated using the literature data [18,44,45] for the most popular apple cultivars (Golden Delicious, Fuji, and Jonagold) in comparison with our data on Piedmont region cultivars. It is noteworthy that among three different studies of the same apple cultivars, there is a lack of agreement regarding sugar content. The studies only agree on the fact that fructose seemed to be the most abundant in all apple cultivars, whereas the relative content of glucose and sucrose was highly variable. The fructose/glucose ratio was always higher than 1.7 for all apple cultivars, whereas the upper limit can be as high as 15 (calculated for Fuji according to literature data [44]). In the case of Piedmont cultivars, the fructose/glucose ratio was in the range of 4.3 (A9)–1.8 (A2). The fructose/sucrose ratio was less variable, ranging from 1.1 (Jonagold, [44]) to 3.0 (Fuji, [45]). The corresponding values for Piedmont cultivars are inside this range, except for one (6.5), corresponding to cultivar A5 with the lowest sucrose content and a high fructose level.

The sugar-to-acid ratio is another important parameter indicating the taste and flavor of apples. In particular, a high level of the sugar/acid ratio is related to higher sweetness [48,49]. Again, the literature data on the sugar-to-acid ratio in Golden Delicious, Fuji, and Jonagold are not consistent—see Table 4. For example, for the Jonagold cultivar, values from 31 [18] to 127 [45] were reported. In our study, the cultivars with the highest sugar/acid ratio were Canditina (A1), Grigia di Torriana (A2), and Carla (A5) due to a low level of total organic acid, whereas a lower sugar/acid ratio was observed for the Dominici (A8), Calvilla (A9), and Grenoble (A10) cultivars.

The content of chlorogenic acid, one of the most abundant polyphenols in apple, has been reported to range from 34.9 (Golden Delicious) to 39.4 mg/100 g of DW (Jonagold) in the pulp of commercial cultivars [45]. Almost all Piedmont cultivars (except A3 and A10) were generally characterized by a higher content of chlorogenic acid with the maximum observed in Gamba Fina A6 (89 mg/100 g DW). 

The fatty acid composition observed in the analyzed apple cultivars was similar to that of the literature data [18]. In particular, DUFA was by far the main class of fatty acids, followed by saturated fatty acids, MUFA, and TUFA. A high percentage of DUFA has been reported for Golden Delicious and Jonagold (more than 50 mol %) [11], whereas among the Piedmont cultivars, the highest DUFA content (45 mol%) was observed in Canditina A1. 

The identification of some characteristic metabolites of apples, such as phloridzin/phloretin, rhamnitol, and citramalic acid, is particularly noteworthy. [26,33,50,51] Their content is largely variable both in the analyzed cultivars and in others described in the literature [18,25,26,52]. These molecules are also widely studied to assign their role in the human body and in apple biosynthetic pathways. 

Phloridzin as a polyphenol compound with antioxidant activity [53] shows some health benefits; in particular, it can be useful in the prevention of the type 2 diabetes mellitus [54,55] by reducing intestinal sugar uptake. Rhamnitol has been considered as a dietary biomarker in relation to the consumption of apples [56] and also as an important metabolite for the geographical discrimination of apple varieties with different geographical origins [26]. Finally, citramalic acid has been studied for its contribution to the development of anthocyanin in apple skin [57] and for its correlation with the storage of apples [58].

## 5. Conclusions

The presented results show that every local variety has its own chemical profile responsible for the sensorial, nutritional, and health-related properties, and they may be used as sources of specific substances that are utilized as ingredients of health products, such as food supplements, functional foods, and cosmetics. For instance, the Gamba Fina (A6) cultivar showed a significant content of chlorogenic acid with recognized properties against metabolic syndrome disorder [59,60], and the Grigia di Torriana (A2), Carla (A5), and Grenoble (A10) cultivars, with their high content of phloretin and phloridzin, could be considered very interesting for their possible properties against insulin resistance [54,55,61]. From a nutritional point of view, being rich in sugars, the Magnana cultivar (A3) can be used to prepare apple juice with no added sugars. Moreover, as the morphological characteristics (especially of the medium/small fruits) make these apple cultivars unattractive to the market for direct consumption, most of them could be successfully employed for the preparation of ingredients of nutraceutical products with high added value.

Although further studies should be performed to gain further understanding of the genetic and environmental basis that leads to these peculiar chemical compositions and the accumulation of polyphenols or nutrients, the reported data could be useful for national and international information systems to reinforce food and agriculture sectors, giving producers and industries accurate information regarding local food peculiarities [62].

## Figures and Tables

**Table 1:** Characteristics (maturity time, peel, pulp and size) of the ten investigated apple cultivars [7,8,9,34].

N°	Cultivar	Maturity Time	Peel	Pulp	Size
A1	Canditina	Late October	Smooth, green with red overcolour	Juicy, white/green	Medium
A2	Grigia di Torriana	Mid/late October	Rough, green	Soft, white	Medium
A3	Magnana	Late October/early November	Smooth/rough, white/green with red overcolour	Soft, white/green	Medium
A4	Runsé	Late October/early November	Smooth, red	Juicy, white/pink	Medium
A5	Carla	Mid/late September	Smooth, yellow with red/orange overcolour	Soft, white	Medium/small
A6	Gamba Fina	Early/Mid October	Smooth, yellow/green with red overcolour	Soft, white	Medium/small
A7	Ross Giambon	Mid October	Smooth, yellow/green	Juicy, white	Large
A8	Dominici	Mid October	Rough, yellow/green with red overcolour	Crisp, white	Large
A9	Calvilla	Mid October	Smooth, green with red overcolour	Crisp and juicy, white/green	Medium
A10	Grenoble	Late October	Rough, green	Crisp, white/green	Small

**Table 2:** Metabolites identified in the 600.13 MHz proton nuclear magnetic resonance (^1^H NMR), ^1^H-^1^H TOCSY, ^1^H-^13^C HSQC, and ^1^H-^13^C HMBC spectra of Bligh–Dyer hydroalcoholic extracts of apple in 0.7 mL of phosphate buffer/D_2_O at 28 °C. Asterisks (*) indicate signals selected for integration.

Compound	Assignment	^1^H (ppm)	Multiplicity (J(Hz))	^13^C (ppm)
*Carbohydrates*				
α-D-Fructofuranose	C-2			105.5
	CH-3	4.13 *		83.0
	CH-4	4.00		77.2
	CH-5	4.07		82.4
β-D-Fructofuranose	C-2			102.6
	CH-3	4.12 *		76.6
	CH-4	4.12 *		75.5
	CH-5	3.84		81.7
	CH2-6,6′	3.83; 3.68		
β-D-Fructopyranose	CH_2_-1,1′	3.57; 3.72		64.9
	C-2			99.2
	CH-3	3.80		68.6
	CH-4	3.90		70.7
	CH-5	4.01		70.2
	CH_2_-6,6′	3.72; 4.03		64.5
α-Xylose	CH-1	5.20 *	d ^a^ (3.8)	93.4
	CH-2	3.55		
	CH-3	3.65		
β- Xylose	CH-1	4.59 *	d (8.0)	97.7
	CH-2	3.54		
	CH-3	3.69		
α-Glucose	CH-1	5.24 *	d (3.8)	93.2
	CH-2	3.55		72.2
	CH-3	3.73		73.7
	CH-4	3.42		70.3
	CH-5	3.84		72.8
β-Glucose	CH-1	4.66 *	d (8.0)	97.0
	CH-2	3.25	dd ^b^ (9.4; 7.9)	75.1
	CH-3	3.51		76.9
	CH-4	3.43		70.7
	CH-5	3.49		76.9
	CH_2_-6,6′	3.90; 3.75		61.8
Sucrose	CH-1 (glucose)	5.42 *	d (3.8)	93.3
	CH-2	3.57		72.0
	CH-3	3.77		73.7
	CH-4	3.47		70.1
	CH-5	3.85		73.5
	C-2 (fructose)			104.8
	CH-3	4.22	d (8.8)	77.3
Rhamnitol	CH_3_	1.28 *	d (6.4)	20.0
	CH-2	3.88		68.2
	CH-3	3.61		74.3
*Organic acids*				
Acetic acid	α-CH_3_	1.92 *	s ^c^	
Citric acid	α,γ-CH	2.54 *	d (15.2)	46.7
	α’,γ’-CH	2.67		46.7
	β-C			76.5
	1,5-COOH			180.8
	6-COOH			183.0
Formic acid	HCOOH	8.46 *	s	
Citramalic acid	β-CH_3_	1.33 *	s	26.5
	β-CH_2_	2.44	d (15.8)	47.7
	β’-CH_2_	2.74	d (15.8)	47.7
	α-C			75.5
	1,4-COOH			184.2
Lactic acid	β-CH_3_	1.33 *	d (7.0)	21.4
	α-CH	4.12		69.8
	COOH			183.4
Malic acid	α-CH	4.30 *	dd (9.9; 3.2)	71.6
	β-CH	2.67	dd (15.4; 3.2)	43.9
	β’-CH	2.37	dd (15.4; 9.9)	43.9
Quinic acid	C-1			78.3
	CH_2_-2, 2′	1.88 *; 2.06	dd (13.5; 10.8); m	41.9
	CH-3	4.03	m ^d^	68.0
	CH-4	3.57	m	76.2
	CH-5	4.16	m	71.3
α-Galacturonic acid	CH-1	5.31	d (3.8)	93.1
	CH-2	3.80		
	CH-3	3.90		
	CH-4	4.29		
	CH-5	4.41*	d (1.2)	72.5
	COOH			177.1
*Amino acids*				
Alanine	α-CH	3.80		51.5
	β-CH_3_	1.49 *	d (7.3)	17.3
	COOH			176.8
Asparagine	α-CH	4.05		52.3
	β,β’-CH_2_	2.88 *; 2.96	dd (7.4; 16.9); dd (4.3; 12.6)	35.6
	COOH			175.5
Aspartate	β,β’-CH_2_	2.70; 2.81 *	dd(3.7; 17.4)	
γ-Aminobutyrate	α-CH_2_	2.30 *	t ^e^ (7.4)	35.3
	β-CH_2_	1.90		24.7
	γ-CH_2_	3.01		
Isoleucine	γ-CH_3_	1.01 *	d (7.1)	
Valine	γ-CH_3_	0.99 *	d (7.1)	
	γ’-CH_3_	1.05	d (7.1)	
*Miscellaneous metabolites*				
Chlorogenic acid	CH_2_-2	2.20		
	CH-3	5.33	m	72.2
	CH_2_-6	2.04		
	CH-1′	6.42 *	d (16.0)	115.8
	CH-2′	7.67	d (16.0)	147.3
	CH-3′	7.22	d (2.0)	116.3
	CH-6′	6.98		116.7
	CH-7′	7.15		123.8
	CH_2_-6, 6′	1.97;2.05	m	38.5
Choline	N(CH_3_)_3_+	3.21 *	s	55.1
*p*-Coumaric acid derivative	CH-2,6	7.62 *	d (8.8)	131.9
	CH-3,5	6.97		
	CH=CH	7.79; 6.51	d (16.1)	
Phloretin/Phloridzin	CH-2,6	7.16	d (8.3)	130.7
	CH-3,5	6.85 *	d (8.3)	116.5
	CH-3′,5′	6.19; 6.24	s	96.6; 97.3
	β-CH_2_	2.92		31.0

^a^ d = doublet; ^b^ dd = double doublet; ^c^ s = singlet; ^d^ m = multiplet; ^e^ t = triplet.

**Table 3:** Metabolites identified in the 600.13 MHz ^1^H NMR, ^1^H-^1^H TOCSY, ^1^H-^13^C HSQC, and ^1^H-^13^C HMBC spectra (28 °C) of Bligh–Dyer organic extracts of apple in CDCl_3_/CD_3_OD (2:1 *v*/*v*) mixture at 28 °C. Asterisks (*) indicate signals selected for integration. For the integration of total fatty acids (I_FA_), the region of 2.22–2.35 was considered. For the integration of total unsaturated fatty acids (I_UNS_), the region of 5.25–5.384 was considered.

Compound	Assignment	^1^H (ppm)	Multiplicity (J(Hz))	^13^C (ppm)
Oleic fatty chain	COO			174.4
(C18:1 Δ^9^)	CH_2_-2	2.30		34.6
	CH_2_-3	1.58	m ^a^	25.3
	CH_2_-4,7	1.30	m	29.5
	CH_2_-8	2.01	m	27.6
	CH=CH 9,10	5.31	m	130.6
	CH_2_-11	2.01	m	27.6
	CH_2_-12,15	1.33–1.30	m	29.4–30.2
	CH_2_-16	1.28	m	31.6
	CH_2_-17	1.26	m	23.0
	CH_3_-18	0.84	t ^b^	14.4
Linoleic fatty chain	COO			174.4
(C18:2 Δ^9,12^)	CH_2_-2	2.30		34.6
	CH_2_-3	1.58	m	25.3
	CH_2_-4,7	1.32–1.28	m	29.5
	CH_2_-8	2.02	m	27.6
	CH= 9	5.34	m	130.6
	CH= 10	5.31	m	128.6
	CH_2_-11	2.73 * (I_DI_)	t (6.8)	26.0
	CH= 12	5.31	m	128.6
	CH= 13	5.34	m	130.6
	CH_2_-14	2.02	m	27.6
	CH_2_-15	1.29	m	29.4
	CH_2_-16	1.29	m	31.6
	CH_2_-17	1.23	m	23.0
	CH_3_-18	0.85	t	14.4
Linolenic fatty chain	COO			174.4
(C18:3 Δ^9,12,15^)	CH_2_-2	2.30		34.9
	CH_2_-3	1.58	m	25.3
	CH_2_-4,7	1.30	m	29.5
	CH_2_-8	2.03	m	27.6
	CH= 9	5.34	m	130.6
	CH= 10	5.30	m	128.6
	CH_2_ 11	2.77 * (I_TRI_)	t (6.2)	26.0
	CH=CH 12,13	5.30	m	128.6
	CH_2_-14	2.77 * (I_TRI_)	t (6.2)	26.0
	CH= 15	5.28	m	127.4
	CH= 16	5.34	m	132.2
	CH_2_-17	2.03	m	20.9
	CH_3_-18	0.94	t (7.6)	14.4
Saturated fatty acids	COO			174.4
	CH_2_-2	2.28		34.6
	CH_2_-3	1.58	m	25.3
	CH_2_	1.28–1.22	m	29.6-32.0
	CH_2_ n-1	1.26		22.9
	CH_3_ n	0.84	t	14.4
Diacylglycerol moiety	CH-*sn* 2	5.06		72.5
	CH-*sn* 1	4.15, 4.33		62.5
	CH-*sn* 3	3.65		61.0
β-Sitosterol	CH_3_-18	0.66 * (I_STE_)	s ^c^	12.2
Squalene	CH_3_-a	1.56		16.3
	CH_3_-b	1.64		25.8
	CH-c	5.07	m	124.8
	CH_2_-d	2.02		27.4
	CH_2_-e	1.96		40.1
1,2-Diacyl-*sn*-glycero-3- phosphatidylcholine	N(CH_3_)_3_+	3.22 * (I_PC_)	s	54.5
	CH_2_N+	3.75		66.4
	CH_2_OP	4.45		60.6
	CH-*sn* 2	5.06		72.5
	CH-*sn* 1	4.15, 4.33		62.5
	CH-*sn* 3	3.65		61.0
Digalactosyldiacylglycerol	CH’’-1	4.87 * (I_DGDG_)	d ^d^(3.8)	99.8
	CH’’-2	3.77		69.2
	CH’’-3	3.69		70.6
	CH’’-4	3.91		70.2
	CH’-1	4.19		104.5
	CH’-2,3	3.51–3.53		
	CH’-4	3.90		
	CH-*sn* 2	5.06		72.5
	CH-*sn* 1	4.15, 4.33		62.5
	CH-*sn* 3	3.65		61.0

^a^ m = multiplet; ^b^ t = triplet; ^c^ s = singlet; ^d^ d = doublet.

**Table 4:** Fructose/glucose, fructose/sucrose, and sugar/acid weight ratios in the ten studied Piedmont region apple cultivars and in three commercial cultivars (calculated using literature data).

Cultivar	Reference	Fru/Glc	Fru/Suc	Sugar/Acid
Golden Delicious	[18]	2.0	2.3	34
Golden Delicious	[44]	2.9	1.8	
Golden Delicious	[45]	3.6	1.9	60
Fuji	[18]	1.7	2.8	42
Fuji	[44]	15.0	2.0	
Fuji	[45]	1.9	3.0	90
Jonagold	[18]	1.7	2.6	31
Jonagold	[44]	7.3	1.1	
Jonagold	[45]	3.2	1.8	127
A1	Present work	2.3	2.4	57
A2	Present work	1.8	3.2	61
A3	Present work	3.6	1.3	44
A4	Present work	3.5	3.1	43
A5	Present work	2.9	6.5	69
A6	Present work	4.2	2.0	42
A7	Present work	3.6	2.1	49
A8	Present work	3.8	2.3	37
A9	Present work	4.3	1.5	37
A10	Present work	3.2	1.3	38

## Data Availability

The data presented in this study are available on request from the corresponding author.

## Supplementary Materials

The following are available online at https://www.mdpi.com/2304-8158/10/2/289/s1, Table S1: Metabolite content in the hydroalcoholic extract of the analyzed apple cultivars from the Piedmont region (mg/100 g ± SD); Table S2: Metabolite content in the organic extract of the analyzed apple cultivars from the Piedmont region (molar % ± SD).
