# Supplementary material for: NMR Characterization of Ten Apple Cultivars from the Piedmont Region
Source: Foods. 2021 Feb 1;10(2):289. doi: 10.3390/foods10020289 (PMC7912530; doi:10.3390/foods10020289)
Supplement: Supplementary file 1 [file foods-10-00289-s001.pdf]

NMR Characterization of Ten Apple Cultivars from the Piedmont Region

Giacomo Di Matteo [1], Mattia Spano [1], Cristina Esposito [2], Cristina Santarcangelo [2], Alessandra Baldi [3], Maria Daglia [2,4], Luisa Mannina [1], Cinzia Ingallina [1,*] and Anatoly P. Sobolev [5]

1 Department of Chemistry and Technology of Drugs, Sapienza University of Rome, Piazzale Aldo Moro 5, 00185 Rome, Italy; giacomo.dimatteo@uniroma1.it (G.D.M.); mattia.spano@uniroma1.it (M.S.); luisa.mannina@uniroma1.it (L.M.); cinzia.ingallina@uniroma1.it (C.I.)

2 Department of Pharmacy, University of Naples Federico II, Naples, 80138, Italy; cristina.esposito@unina.it (C.E.); cristina.santarcangelo@unina.it (C.S.)

3 Tefarco Innova, Parco Area delle Scienze 27/A – Campus 43124 Parma, Italy; alessandra.baldi.alimenti@gmail.com (A.B.)

4 International Research Center for Food Nutrition and Safety, Jiangsu University, 212013 Zhenjiang, China; maria.daglia@unina.it (M.D.)

5 Institute for Biological Systems, Magnetic Resonance Laboratory "Segre-Capitani", CNR, Via Salaria Km 29.300, 00015 Monterotondo (Rome), Italy; anatoly.sobolev@cnr.it (A.S.)

* Correspondence: cinzia.ingallina@uniroma1.it

Table S1. Metabolite content in the hydroalcoholic extract of the analyzed apple cultivars from the Piedmont region (mg/100g ± SD).

	A1 Canditina	A2 Grigia di Torriana	A3 Magnana	A4 Runsé	A5 Carla	A6 gamba Fina	A7 Ross Giambon	A8 Dominici	A9 Calvilla	A10 Grenoble
Valine	0.17 ± 0.01	0.39 ± 0.03	2.45 ± 0.20	0.67 ± 0.04	0.35 ± 0.02	0.19 ± 0.01	0.31 ± 0.02	0.57 ± 0.04	0.27 ± 0.02	1.10 ± 0.09
Isoleucine	0.54 ± 0.04	0.38 ± 0.02	7.26 ± 0.51	1.39 ± 0.08	0.39 ± 0.03	0.49 ± 0.03	0.33 ± 0.02	3.59 ± 0.25	1.22 ± 0.10	0.64 ± 0.03
Rhamnitol	54.65 ± 5.11	37.35 ± 3.12	39.51 ± 3.13	26.40 ± 2.15	28.87 ± 2.64	44.45 ± 3.22	25.89 ± 1.45	65.79 ± 3.22	37.44 ± 1.56	76.53 ± 4.21
Alanine	1.11 ± 0.07	6.24 ± 0.25	11.37 ± 0.89	6.45 ± 0.61	1.82 ± 0.09	2.16 ± 0.07	3.67 ± 0.21	5.73 ± 0.51	3.93 ± 0.27	11.28 ± 0.91
Quinic acid	54.60 ± 4.31	137.69 ± 11.52	47.79 ± 3.28	84.53 ± 6.67	254.62 ± 19.84	83.02 ± 7.02	76.20 ± 5.42	37.58 ± 3.63	81.33 ± 7.12	73.25 ± 7.4
Acetic acid	5.03 ± 0.76	5.17 ± 0.65	4.47 ± 0.69	5.94 ± 0.87	6.85 ± 1.05	5.50 ± 0.84	5.48 ± 0.91	5.18 ± 0.83	5.87 ± 0.72	4.47 ± 0.69
GABA	/	2.91 ± 0.19	9.40 ± 0.78	2.15 ± 0.19	1.12 ± 0.08	2.15 ± 0.15	2.68 ± 0.23	5.13 ± 0.49	3.43 ± 0.35	7.08 ± 0.71
Citric acid	21.45 ± 1.91	49.33 ± 3.52	40.81 ± 3.27	52.24 ± 3.98	29.62 ± 1.87	28.34 ± 2.44	37.53 ± 3.29	29.56 ± 2.11	65.26 ± 4.39	48.71 ± 3.88
Aspartate	/	15.08 ± 1.32	35.74 ± 3.17	16.47 ± 1.28	6.29 ± 0.53	/	/	33.04 ± 3.23	14.33 ± 1.14	28.21 ± 2.24
Asparagine	/	59.23 ± 4.49	93.71 ± 8.57	63.43 ± 6.58	16.16 ± 1.17	39.24 ± 2.69	7.25 ± 0.59	226.95 ± 17.25	36.29 ± 2.56	285.55 ± 23.61
Choline	0.98 ± 0.07	2.14 ± 0.15	2.19 ± 0.16	1.06 ± 0.07	1.90 ± 0.23	1.42 ± 0.09	1.32 ± 0.14	1.38 ± 0.06	0.90 ± 0.04	1.41 ± 0.12

Fructose	20499.69 ± 1755.12	31386.13 ± 2639.23	28810.47 ± 2433.54	32426.44 ± 2541.36	34047.56 ± 3125.75	21983.33 ± 1752.65	34637.15 ± 3468.28	29327.38 ± 2544.14	31644.41 ± .2659.31	26224.20 ± 1923.47
Malic acid	444.81 ± 39.31	514.49 ± 43.19	1051.52 ± 93.26	904.33 ± 84.52	354.54 ± 31.27	567.94 ± 59.44	915.97 ± 75.27	1126.27 ± 96.51	1249.83 ± 98.63	1248.81 ± 85.69
Sucrose	8613.12 ± 621.23	9936.07 ± 634.28	21846.88 ± 19	10536.16 ±	5274.83 ±	10827.21 ±	16661.66 ±	12558.56 ±	20906.13 ±	20661.58 ±
Chlorogenic acid	65.77 ±	77.97 ±	16.93 ±	58.53 ±	40.83 ±	89.67 ±	54.29 ±	46.40 ±	66.54 ±	23.90 ±
Formic acid	1.07 ± 0.17	1.41 ± 0.18	1.23 ± 0.19	1.49 ± 0.13	1.78 ± 0.24	1.12 ± 0.16	0.73 ± 0.10	1.02 ± 0.15	0.88 ± 0.14	1.01 ± 0.17
Lactic acid	19.28 ± 1.72	29.10 ± 2.21	24.59 ± 2.11	41.06 ± 4.01	39.43 ± 2.96	35.38 ± 3.32	29.91 ± 2.11	41.82 ± 3.52	34.41 ± 3.61	14.26 ± 1.15
Citramalic acid	51.67 ± 4.46	12.14 ± 1.12	34.69 ± 3.34	34.87 ± 3.14	/	10.83 ± 0.85	36.21 ± 3.65	21.15 ± 2.02	32.20 ± 2.83	5.83 ± 2.31
Glucose	8760.26 ± 123.48	17415.56 ± 1534.52	8053.86 ± 405.23	9380.45 ± 684.36	11623.71 ± 1254.32	5262.43 ± 433.27	9692.63 ± 424.65	7740.80 ± 294.33	7433.31 ± 711.25	8128.47 ± 358.24
Xylose	97.40 ± 8.51	302.07 ± 23.52	605.23 ± 45.26	207.70 ± 11.23	630.24 ± 55.94	542.05 ± 56.23	447.79 ± 32.36	530.12 ± 41.69	314.08 ± 17.85	281.77 ± 11.23
Galacturonic acid	/	139.22 ± 9.54	113.29 ± 8.14	51.64 ± 4.56	18.92 ± 1.32	105.88 ± 7.23	90.81 ± 7.71	61.37 ± 3.96	105.47 ± 10.78	53.38 ±1.92
Phloretin equivalent[a]	5.45 ± 0.32	42.87 ± 3.88	5.67 ± 1.29	6.74 ± 4.63	32.68 ± 3.11	6.70 ± 2.25	7.37 ± 0.69	7.81 ± 0.15	5.54 ± 0.49	31.73 ± 1.26

p-coumaric acid equivalent [b]	9.39 ± 0.45	6.45 ± 0.12	3.13 ± 0.33	3.89 ± 0.29	2.34 ± 0.15	2.53 ± 0.14	4.01 ± 0.26	1.61 ± 0.12	1.67 ± 0.15	1.12 ±0.09

[a] Phloretin/phloridzin is expressed as mg of phloretin equivalents/100 g.

[b] *p*-Coumaroyl derivative is expressed as mg of coumaric acid equivalents/100 g.

Table S2. Metabolite content in the organic extract of the analyzed apple cultivars from the Piedmont region (molar % ± SD).

	A1 Canditina	A2 Grigia di Torriana	A3 Magnana	A4 Runsé	A5 Carla	A6 gamba Fina	A7 Ross Giambon	A8 Dominici	A9 Calvilla	A10 Grenoble
TOT FA	87.97 ± 8.41	82.36 ± 4.56	86.36 ± 3.26	86.92 ± 3.78	86.73 ± 4.52	87.61 ± 8.12	87.09 ± 4.63	85.69 ± 2.74	87.07 ± 3.56	84.80 ± 4.48
DUFA	30.92 ± 2.88	44.97 ± 3.26	36.32 ± 1.96	39.50 ± 2.56	33.40 ± 3.20	32.23 ± 1.15	36.01 ± 2.89	39.67 ± 1.98	41.55 ± 2.54	39.29 ± 3.12
TUFA	10.81 ± 0.95	4.45 ± 0.29	3.52 ±0.15	3.22 ± 0.18	3.40 ± 0.29	4.49 ± 0.51	3.68 ± 0.18	3.88 ± 0.26	5.60 ± 0.43	7.68 ± 0.35
PC	15.18 ± 1.29	13.82 ± 1.14	15.64 ± 1.36	16.88 ± 1.28	13.37 ± 0.86	13.80 ± 0.85	17.69 ± 1.56	20.22 ± 1.43	22.32 ± 2.13	23.79 ± 1.59
DGDG	3.99 ± 0.26	5.46 ± +0.36	2.46 ± 0.18	4.59 ± 0.14	4.18 ± 0.13	2.67 ± 0.11	2.77 ± 0.21	2.84 ± 0.23	4.66 ± 0.36	4.36 ±0.29
TOT UFA	47.76 ± 3.62	59.96 ± 4.36	53.97 ± 3.26	66.32 ± 4.21	51.62 ± 2.69	59.58 ± 5.32	61.63 ± 4.59	66.08 ± 3.69	61.54 ± 2.54	63.77 ± 1.69

MUFA	6.03 ± 0.46	10.55 ± 0.65	14.13 ± 0.94	23.60 ± 0.96	14.81 ± 1.12	22.85 ± 1.36	21.94 ± 0.86	22.53 ± 1.36	14.38 ± 0.96	16.81 ± 1.52
TOT SFA	40.20 ± 2.36	22.40 ± 1.23	32.39 ± 2.36	20.60 ± 1.21	35.12 ± 2.41	28.04 ± 1.29	25.45 ± 2.23	19.61 ± 1.34	25.53 ± 2.36	21.03 ± 1.39
